# Optimization of ultrasonic-assisted extraction of flavonoids from *Lactuca indica* L. cv. Mengzao and their antioxidant properties

**DOI:** 10.3389/fnut.2023.1065662

**Published:** 2023-06-15

**Authors:** Junfeng Hao, Zhijun Wang, Yushan Jia, Lin Sun, Zhihui Fu, Muqier Zhao, Yuyu Li, Ning Yuan, Baiming Cong, Lixing Zhao, Gentu Ge

**Affiliations:** ^1^Key Laboratory of Forage Cultivation and the Processing and High Efficient Utilization of the Ministry of Agriculture, Key Laboratory of Grassland Resources of the Ministry of Education, College of Grassland, Resources and Environment, Inner Mongolia Agricultural University, Hohhot, China; ^2^Inner Mongolia Academy of Agricultural & Animal Husbandry Sciences, Hohhot, China; ^3^Institute of Grassland Research, Chinese Academy of Agricultural Sciences, Hohhot, China; ^4^Tongliao Agricultural and Animal Husbandry Science Research Institute, Tongliao, China; ^5^Hinggan League Agricultural and Animal Husbandry Science Research Institute, Ulanhot, China

**Keywords:** *Lactuca indica* L. cv. Mengzao, flavonoids, ultrasound-assisted extraction, UPLC–MS, antioxidant activity

## Abstract

In this study, the ultrasound-assisted extraction (UAE) conditions of flavonoids from *Lactuca indica* L.cv. Mengzao (LIM) leaves was optimized, and the flavonoids content and their antioxidant potential in different parts were analyzed. The optimal extraction parameters to obtain the highest total flavonoids content (TFC) were a a ratio of liquid to solid of 24.76 mL/g, ultrasonic power of 411.43 W, ethanol concentration of 58.86% and an extraction time of 30 min, the average TFC of LIM leaves could reach 48.01 mg/g. For the yield of flavonoids, the UAE method had the best extraction capacity compared with solvent extraction and microwave-assisted extraction (MAE). In general, the TFC in different parts of LIM followed the order flower > leaf > stem > root, the flowering period is the most suitable harvesting period. From ultra-high performance liquid chromatography-mass spectrometry (UPLC–MS) quantification, the flower samples showed significantly higher six flavonoids and had the highest radical scavenging capacities compared to other samples. A high positive correlation was observed between the antioxidant activity and TFC, luteolin-7-O-glucoside and rutin were significantly (*p* < 0.05) correlated with all antioxidant evaluations. This study provides valuable information for the development and utilization of flavonoids in *Lactuca indica* as ingredients in food, feed and nutritional health products.

## Introduction

Considering that the use of herbal resources for nutraceutical purposes is now intensively promoted on the basis of both ethnopharmacological and scientific evidence, research in this field is constantly growing (1). In China, herbal medicine has been used in medical care for thousands of years. *Lactuca indica* is an annual or perennial herbs of the Compositae family. It grows mainly in the mountains. When the stem and leaf are cut, they produce a bitter lotion-like juice. This is why it is also called bitter cabbage. It was first recorded as an elegant plant in *Shennong Bencao Jing*, and as a medicine in *Neimenggu Traditional Chinese Medicine*. It is widely distributed in many provinces and areas of China, such as Shandong, Hebei, Neimenggu and Northeast etc. (2). The roots and leaves of *Lactuca indica* have been eaten as wild vegetables in China since ancient times. They are widely used in folk therapy for sedation, hypnosis, antipyretic, hematopoietic and digestive purposes (2, 3). *Lactuca indica* contains various physiologically active substances such as alphatics, flavonoids, triterpenes, sesquiterpene lactones (2). In addition, a number of research results have shown that the root of *Lactuca indica* has anticancer activity, blood lipid lowering, blood sugar lowering and antioxidant activities, suggesting that the extract of bitter cabbage root may contain antioxidant and effective physiologically active substances that inhibit cancer cells (4–6). In recent years, there have been an increasing number of studies on the development of health functional materials using *Lactuca indica*, but research on the use of its leaf, stem and flower as health functional materials has not been pursued. In addition, there have been few studies on the physiological activity of the leaf, stem and flowerof *Lactuca indica* after harvesting.

In general, the physiological functions of herbs are strongly dependent on the composition of active constituents and their content (7). In particular, flavonoids are the main components of *Lactuca indica*. However, it has been reported that the content of flavonoids in *Lactuca indica* was 1.66–4.76% by the differernt extraction methods (8). It is necessary to further optimise the extraction process for quality control. Extraction is the key step in the determination and quantification of active constituents in plant material. The extraction process separates the bioactive and functional compounds from various herbal raw materials and natural sources (9).

Conventional methods such as Soxhlet extraction, heating reflux extraction and other extraction methods have been used to extract flavonoids from plants (10, 11). Although the above methods are simple to use, there are also some practical problems, such as the long extraction times, high levels of impurities, and low product purity and low extraction rates (12). In recent years, advanced methods, including ultrasonic-assisted extraction (UAE), microwave-assisted extraction (MAE) and enzyme-assisted extraction (EAE) have been developed and widely used for the extraction of plant flavonoids, polysaccharides, alkaloids and volatile oils because they can significantly reduce the damage to the target compounds, have high yields and use less solvent (13). Previous literatures have compared the flavonoid yields of different extraction methods for the extraction of flavonoids from *Lactuca indica*. According to their results (8, 14), the new methods always have a higher flavonoid content or are less time-consuming than the conventional methods (13). However, to the best of our knowledge, there is no available literature reporting on the extraction of flavonoids from *Lactuca indica* L. cv. Mengzao (LIM) using UAE technologies. Furthermore, there are limited studies on the relationship between the bioactivities of flavonoids and the spatial and temporal distribution of LIM.

In this study, we aimed to investigate UAE in detail in order to fit the kinetic model of UAE extraction, optimise the extraction process of UAE, and reduce the extraction cost. Meanwhile, we evaluated the effects of the extraction process on the total flavonoids content (TFC) of LIM leaves and investigated the relationship between antioxidant activity and flavonoids. In the first part of this study, we took TFC as the primary variable and focused on the optimisation of TFC process parameters, considering liquid–solid ratio, ultrasonic power, ethanol concentration, and extraction time as optimisation parameters. Box–Behnken design (BBD) followed by canonical and ridge analyses were used to optimise the process parameters of TFC from LIM leaves. In the second part, the sensitive and fast ultra–high performance liquid chromatography mass spectrometry (UPLC–MS) method was used to simultaneously quantify six flavonoids, including luteolin, rutin, quercetin, luteolin-7-O-glucoside, apigenin and kaempferolin from different parts of LIM. Finally, the relationship between antioxidant activity and flavonoids was investigated. This study may provide an efficient alternative and “green” technique for the deep processing of flavonoids from *Lactuca indica*.

## Materials and methods

### Plant materials

Materials tested LIM were planted in the pasture base of Inner Mongolia Agricultural University on May 10, 2020. Organic fertilizer (sheep manure fermentation) was previously applied to the field. Materials were watered and weeded, and sampled from July to September. The materials were divided into roots, stems, leaves and flowers, dried in the shade at room temperature, crushed, and stored for subsequent testing and analysis. Information on the materials is summarized in Table 1.

**TABLE 1:** Harvest time, growth period and harvest parts of LIM.

Harvest time	Growth period	Harvest part
4 July In 2020	Vegetative growth period	Roots, stems, leaves
25 July in 2020	Flowering stage	Roots, stems, leaves, flowers
24 August in 2020	Filling stage	Roots, stems, leaves

### Chemicals and reagents

In this study, most of the chemicals and reagents were analytical grade. Ethanol, ferric chloride, hydrogen peroxide, salicylic acid, sodium carbonate catechin, 2,2′-diphenyl-1-picrylhy-drazyl (DPPH) and 2,2′-azinobis-(3-ethylbenzothiazoline-6-sulfonic acid) (ABTS) were purchased from Sinopharm Chemical Reagent Co. Ltd (Shanghai, China). Chemical standards including rutin (purity > 98%), luteolin (purity > 98%), apigenin (purity > 98%), quercetin (purity > 98%), luteolin-7-O-glucoside (purity > 98%) and kaempferol (purity > 98%) were purchased from Yuanye Biotechnology Co., Ltd. (Shanghai, China). HPLC grade reagents including methanol, acetonitrile and formic acid were purchased from Thermo Fisher Scientific (San Jose, CA, USA).

### Optimization of ultrasound-assisted extraction of flavonoids

#### Ultrasound-assisted extraction of flavonoids

Flavonoids extraction was carried out using an S450 series ultrasonic cleaner (Elma Schmidbauer GmbH, Konstanz, Germany). Samples for the extraction test were selected from LIM leaves at the flowering stage. Powder samples (1.0 g) were placed in an Erlenmeyer flask (150 mL), soaked in a certain volume ethanol solution and then placed in the ultrasonic cleaning bath. To avoid solvent evaporation, the conical flask was covered with parafilm during the extraction process. Different experimental parameters were designed to optimise the extraction process, such as the liquid–solid ratio, ultrasonic power, ethanol concentration and extraction time. After extraction, the crude extract was filtered through a filter paper (120 mm, Whatman^®^, China) and then concentrated at 3,000 rpm for 5 min. The supernatant was collected and diluted with 60 mL of solvent. Each experiment was repeated three times and stored at 4*^o^*C.

#### Determination of TFC

TFC was determined by the method of Yi et al. (15) with some modifications. Briefly, the AlCl_3_-NaOH-NaNO_2_ method was used. 2.0 mL of rutin standard solution with different concentrations (6.25, 12.5, 18.75, 25.0, 31.25, and 37.5 μg/mL) and 0.4 mL of 5% NaNO_2_ solution were mixed for 6 min, and then 0.4 mL of 10% AlCl_3_ solution was shaken and reacted, and 6 min later, 4 mL of 4% NaOH solution and 3.2 mL of distilled water were added and mixed. Distilled water replaced the standard solution as the blank control. After reaction at room temperature for 25 min, the absorbance value was measured at 510 nm using a UV-1900i series Ultraviolet-Visible Spectrophotometer (Shimadzu Co., Ltd, Japan). The calibration curve (*y* = 0.0115*x* – 0.0767, where *y* is the absorbance value of a sample and *x* is the sample concentration), ranged from 6.25 to 37.5 μg/mL (*R*^2^ = 0.9990). 0.2 mL of sample extract was added to solvent to make 2.0 mL, and TFC in the sample extracts was quantified using the standard curve method.

#### Single-factor experiment

The effects of the liquid–solid ratio, ultrasonic power, ethanol concentration and extraction time on TFC from LIM leaves were studied by varying the level of one factor and keeping the other three factors constant. The detailed conditions for each extraction were as follows: when the liquid-solid ratios were 10, 15, 20, 2, and 30 mL/g, the samples were extracted with a 40% ethanol concentration at 250 W for 30 min; when the ultrasonic powers were 150, 250, 350, 450, and 550 W, the samples were extracted with a liquid-solid ratio of 30 g/mL and 40% ethanol concentration for 30 min; when the ethanol concentration were 20, 40, 60, 80, and 99.7%, the samples were extracted with a liquid–solid ratio of 30 g/mL at 250 W for 30 min; when the extraction time were 15, 30, 45, 60, and 75 min, the samples were extracted with a liquid–solid ratio of 30 g/mL and 40% ethanol concentration at 250 W.

#### Optimization experimental design

Based on the preliminary results of the single-factor test, the proper range for each factor was determined, and then a response surface methodology (RSM) was conducted using BBD in Design Expert 8.0.6 software (StatEase^®^, Minneapolis, USA). As shown in Table 2, the four factors chosen for this study were designed as A (liquid–solid ratio), B (ultrasonic power), C (ethanol concentration) and D (extraction time), and were prescribed three levels, coded as -1, 0 and 1. The TFC was taken as the response of the design experiments. The experimental design included 29 trials including 5 replicates of the center point.

**TABLE 2:** BBD and corresponding observed responses.

	Independent variable				
	**A**	**B**	**C**	**D**	
**Test**	**Ratio of liquid to solid (mL/g)**	**Ultrasonic power (W)**	**Ethanol concentration (%)**	**Extraction time (min)**	**Response of total flavonoids content/(mg/g)**
1	–1 (20)	–1 (300)	0 (60)	0 (45)	39.83
2	1 (30)	–1 (300)	0 (60)	0 (45)	38.86
3	–1 (20)	1 (500)	0 (60)	0 (45)	40.04
4	1 (30)	1 (500)	0 (60)	0 (45)	43.49
5	0 (25)	0 (400)	–1 (40)	–1 (30)	45.45
6	0 (25)	0 (400)	1 (80)	–1 (30)	44.75
7	0 (25)	0 (400)	–1 (40)	1 (60)	45.61
8	0 (25)	0 (400)	1 (80)	1 (60)	43.24
9	–1 (20)	0 (400)	0 (60)	–1 (30)	45.32
10	1 (30)	0 (400)	0 (60)	–1 (30)	44.55
11	–1 (20)	0 (400)	0 (60)	1 (60)	43.55
12	1 (30)	0 (400)	0 (60)	1 (60)	45.49
13	0 (25)	–1 (300)	–1 (40)	0 (45)	39.72
14	0 (25)	1 (500)	–1 (40)	0 (45)	41.62
15	0 (25)	–1 (300)	1 (80)	0 (45)	40.37
16	0 (25)	1 (500)	1 (80)	0 (45)	40.55
17	–1 (20)	0 (400)	–1 (40)	0 (45)	44.13
18	1 (30)	0 (400)	–1 (40)	0 (45)	40.94
19	–1 (20)	0 (400)	1 (80)	0 (45)	38.54
20	1 (30)	0 (400)	1 (80)	0 (45)	42.51
21	0 (25)	–1 (300)	0 (60)	–1 (30)	42.81
22	0 (25)	1 (500)	0 (60)	–1 (30)	44.95
23	0 (25)	–1 (300)	0 (60)	1 (60)	42.72
24	0 (25)	1 (500)	0 (60)	1 (60)	44.80
25	0 (25)	0 (400)	0 (60)	0 (45)	47.12
26	0 (25)	0 (400)	0 (60)	0 (45)	46.82
27	0 (25)	0 (400)	0 (60)	0 (45)	47.98
28	0 (25)	0 (400)	0 (60)	0 (45)	47.64
29	0 (25)	0 (400)	0 (60)	0 (45)	48.35

### Other extraction methods

#### Solvent extraction

The extraction parameters were chosen according to the optimum conditions previous obtained by single factorial tests (8). Samples of powder (1.0 g) from LIM leaves were placed in a 500 mL conical flask, and 30 mL of 70% anhydrous ethanol was added to the conical flask. The conical flask was sealed and placed in a water bath at the temperature of 80*^o^*C for 40 min. The extract solvent was filtered and collected in sealed brown reagent bottles for further testing.

#### MAE

The extraction parameters were chosen according to the optimum conditions previous obtained by single factorial tests (8). Samples of powder (1.0 g) from LIM leaves was mixed with 30 mL of 70% anhydrous ethanol. The mixture was sonicated at 30*^o^*C using a microwave extractor (XH-100B, Xianghu, Beijing, China). The treatment time was 60 min. The extract solvent was filtered and collected in brown reagent bottles.

### Determination of antioxidant activity of flavonoids

#### DPPH radical-scavenging assay

The DPPH radical scavenging assay of sample extracts was taken from the reported literature with slight modifications (16). 2.0 mL of 4 mg/mL sample extract solution was mixed with 2.0 mL of 0.1 mM freshly prepared DPPH solution (in methanol). After incubation in the dark for 30 min at room temperature, the absorbance of the different sample extract solutions was measured at 517 nm using a Synergy H1 Hybrid Multimode Microplate Reader (Biotek, USA), using ascorbic acid as a positive control. The DPPH radical scavenging activity was calculated using the following formula:


(1) $$\mathrm{Scavenging}\mathrm{rate}\left(\%\right)=\left[1-\left(\frac{A_{s}-A_{b}}{A_{O}}\right)\right]\times 100$$

where *A_O_* represents the absorbance of the control group (deionized water instead of the flavonoid solution), *A_s_* was the absorbance of the sample solution, and *A_b_* was the absorbance of the sample only, as measured in methanol instead of DPPH solution.

#### ABTS radical-scavenging assay

The ABTS radical scavenging assay of the sample extracts was described in a previous study with slight modifications (17). 80 μL of 4 mg/mL sample solution was mixed with 3.9 mL ABTS working solution. The mixture was allowed to stand for 6 min at room temperature. The absorbance of the different sample extract solutions was measured at 734 nm using a Synergy H1 Hybrid Multimode Microplate Reader (Biotek, USA), using ascorbic acid as a positive control. The ABTS radical scavenging activity was calculated according to Eq. 1, where *A_O_* represents the absorbance of the control group (distilled water instead of the flavonoid solution). *A_s_* is the sample solution absorbance, and *A_b_* is the absorbance of the sample only (deionized water instead of ABTS solution).

#### Hydroxyl radical-scavenging activity

The hydroxyl radical scavenging assay of the sample extracts was described in a previous study with slight modifications (18). 2mL of 4.0 mg/mL sample solution was mixed with 1 mL FeSO_4_ (3 mmol/L), 1 mL of hydrogen peroxide (3 mmol/L), shaken and allowed to standed for 10 min, 1 mL salicylic acid (3 mmol/L) was added, shaken up and allowed to stand for 30 min. Distilled water and ascorbic acid were used as blank and control groups, respectively. The hydroxyl radical scavenging rate was calculated according to Eq. 1, where *A_s_*, *A_b_*, and *A_O_* correspond to the absorbances of the sample solution reaction, the sample only and the deionised water blank group, respectively, all measured at 510 nm.

#### Sample determination

All samples were extracted according to the method described in ultrasound-assisted extraction of flavonoids, and determined according to the method of Hao et al. (3).

#### Statistical analysis

Data were expressed as the mean ± standard deviation of triplicate measurements. Analysis of variance was performed using SPSS 24.0 (SPSS Inc., Chicago, IL, USA). Statistical analysis of the results was carried out using analysis of variance (ANOVA) with Duncan’s test, and statistical significance of differences between the means of each factor were determined at *p* < 0.05. Charts were drawn with OriginPro 2021 (Origin Lab^®^, MA, USA). A correlation clustering marker heatmap was constructed using an online tool (https://www.omicstudio.cn/tool?order=complex). The multivariate statistical analysis including a principal component analysis (PCA), XLSTAT-2019.1.3 were used by Addinsoft Inc. New York, NY, USA.

## Results and discussion

### Optimization of TFC conditions

#### Influence of ratio of liquid to solid on TFC

**FIGURE 1:**
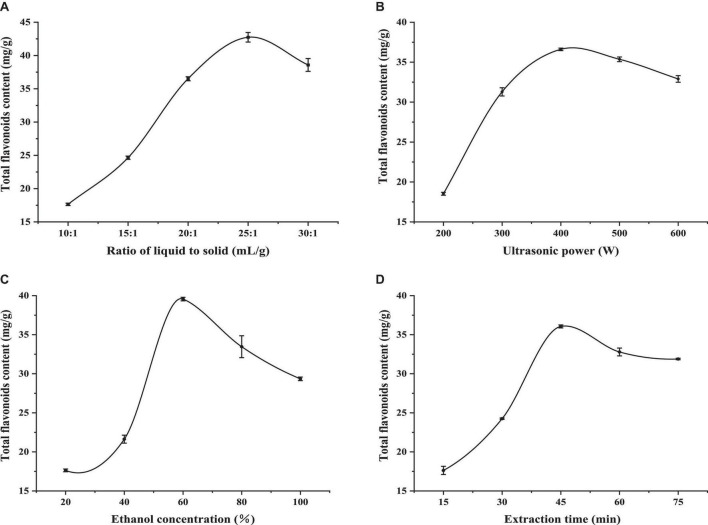
The effects of the ratio of liquid to solid **(A)**, ultrasonic power **(B)**, ethanol concentra-tion **(C)**, and extraction time **(D)** on the extraction content of flavonoids in LIM. Values are expressed as means ± SD (*n* = 3).

The liquid–solid ratio reflects the amount of solvent. In general, the greater the amount of solvent, the greater the extraction rate (19). Figure 1A shows that the TFC of LIM leaves increased rapidly as the liquid–solid ratio increased from 10:1 to 25:1 mL/g. As the liquid–solid ratio increased, the cavitation effect of bubble rupture would be more intense and the mass transfer process would be faster, leading to higher extraction efficiency (20, 21). However, when the liquid–solid ratio increased to 25:1 mL/g, the active components were basically dissolved by a certain proportion of the solvent, so the extraction rate no longer increased, and the amount of ethanol was so large that the dissolution of other impurities also increased, resulting in a decrease in the yield of flavonoids (22). Therefore, the liquid–solid ratio of 20:1∼30:1 mL/g was selected as the range for further optimisation in terms of extraction rate, solvent dosage and production cost.

#### Influence of ultrasonic power on TFC

In order to study the effect of ultrasonic power on extraction performance, the range of ultrasonic power was set from 200 to 600 W and other experimental parameters were set as follows: liquid–solid ratio 15 mL/g, ethanol concentration 40% and ultrasonic 30 min. It can be seen from Figure 1B that ultrasonic power had an important effect on TFC. The TFC of LIM leaves reached a maximum value at 400 W. Above this, a declining tendency was observed. This result was in accordance with a range of previous reports, where TFC increased with ultrasonic power (23, 24). When ultrasonic power was too high, the solvent volatilized and the two flavonoids were destroyed. Therefore, ultrasonic power of 300∼500 W was selected for further optimization.

#### Influence of ethanol concentration on TFC

In general, extraction solvent is a significant parameter for extracting active compounds from plant material. In this study, we weighed a given quality of sorrel powder, added a given concentration of ethanol solution (20, 40, 60, 80, and 99.7%) and extracted flavonoids under these conditions. The results are shown in Figure 1C. It was observed that when the ethanol concentration was 60%, TFC of LIM leaves reached a peak, and above 60%, the amount extracted decreased. TFC first increased and then decreased with an increase in the volume fraction of ethanol, which may be due to the good solubility of ethanol and its strong cell penetration. When the material was treated with a higher volume fraction of ethanol, the concentration difference between inside and outside of tissue cells increased, which was conducive to the dissolution of flavonoids. However, when the volume fraction of ethanol is too high, the dissolution amount of some alcohol-soluble pigments and lipophilic components will increase (25), and these components will compete with ethanol-water molecules to combine, resulting in a decrease in the possibility of flavonoids binding with ethanol-water molecules, causing a decreasing trend in the extraction rate. Therefore, 40∼80% ethanol was used as the reference range for optimization.

#### The influence of extraction time on TFC

The choice of extraction time was another important step to guarantee the distribution equilibrium of flavonoids between sample and extraction solvent (26). The results are shown in Figure 1D. TFC of LIM leaves increased markedly with increasing extraction time from 15 to 45 min. but over 45 min, TFC slightly decreased. With increasing extraction time, the extrac-tion of total flavonoids showed a trend of first increasing and then decreasing. There are two possible reasons for this. In the initial stage of extraction, due to the concentration difference between the two systems of raw material and extraction solution, the TFC increased with increasing extraction time. When a certain time was reached, the flavonoid mass concentration inside and outside the raw material reached a relative balance, and the flavonoid in the raw material no longer continues to easily leach, and the extraction rate tended to be stable. Another reason could be that the extraction of flavonoids is affected by the dissolution of other alcohol-soluble substances as extraction time increases (27). In summary, 30∼60 min was selected for optimization.

### Optimization of ultrasonic-assisted parameters for flavonoids by BBD

#### Model fitting analysis

After these pre-experiments, four main variable levels were determined for the liquid–solid ratio (20:1∼30:1 mL/g), ultrasonic power (300∼500 W), ethanol concentration (40∼80%) and extraction time (30∼60 min). The experimental design and corresponding response data for the TFC of LIM leaves are presented in Table 2. Regression analysis showed that the extraction of TFC was predicted by the second-order equation (Eq. 2).


(2) Y=47.58+0.37⁢A+0.93⁢B-0.62⁢C-0.20⁢D+1.10⁢AB

+1.79⁢AC+0.68⁢AD-0.43⁢BC-0.016⁢BD-0.42⁢CD

-3.05A-23.98B-23.02C+20.20D2

where Y represented the response of the TFC, A represented the solid–liquid ratio, B represented the ultrasonic power, C represented the ethanol concentration and D represented extraction time.

From the ANOVA results in Table 3, it shows that the independent variables (A, B and C), and three quadratic terms (A^2^, B^2^ and C^2^) had significant influences on TFC (*p* < 0.05). Meanwhile, there was also a significant interaction between the liquid–solid ratio and ultrasonic power (AB), between the liquid–solid ratio and ethanol concentration (AC), and between the liquid–solid ratio and extraction time (AD) (*p* < 0.05). For each term in the model, a large *F* value and a small *p*-value indicated a significant effect on the respective response variables (28). The decision coefficient of the model (R^2^) is 0.98, and the adjusted decision coefficient (Adj-R^2^) was 0.96, which showed that the model had a good fit and strong correlation between the predicted results and the actual results (29). The model *p*-value was less than 0.05, indicating that the experimental model was significant. The *p*-value for lack of fit was significant (*p* > 0.05), indicating that the regression equation fitted well in the whole regression area (30).

**TABLE 3:** ANOVA for the response surface model.

Source	Sum of squares	Df	Mean squares	*F* value	*p*-value
					**Prop > *F***
Model	219.00	14	15.64	44.73	<0.0001
A- ratio of liquid to solid	1.64	1	1.64	4.68	0.0484
B-ultrasonic power	10.35	1	10.35	29.60	<0.0001
C-ethanol concentration	4.69	1	4.69	13.40	0.0026
D-extraction time	0.49	1	0.49	1.40	0.2559
AB	4.87	1	4.87	13.93	0.0022
AC	12.82	1	12.82	36.67	<0.0001
AD	1.84	1	1.84	5.27	0.0376
BC	0.75	1	0.75	2.14	0.166
BD	0.00	1	0.00	0.00	0.9583
CD	0.70	1	0.70	1.99	0.1801
A^2^	60.18	1	60.18	172.06	<0.0001
B^2^	102.81	1	102.81	293.97	<0.0001
C^2^	59.28	1	59.28	169.49	<0.0001
D^2^	0.27	1	0.27	0.77	0.3951
Residual	4.90	14	0.35		
Lack of fit	3.35	10	0.34	0.87	0.6130
Pure error	1.55	4	0.39		
Cor total	223.90	28			
*R* ^2^	0.98				
Adj-*R*^2^	0.96				

### Response surface optimization analysis of flavonoid extraction conditions

**FIGURE 2:**
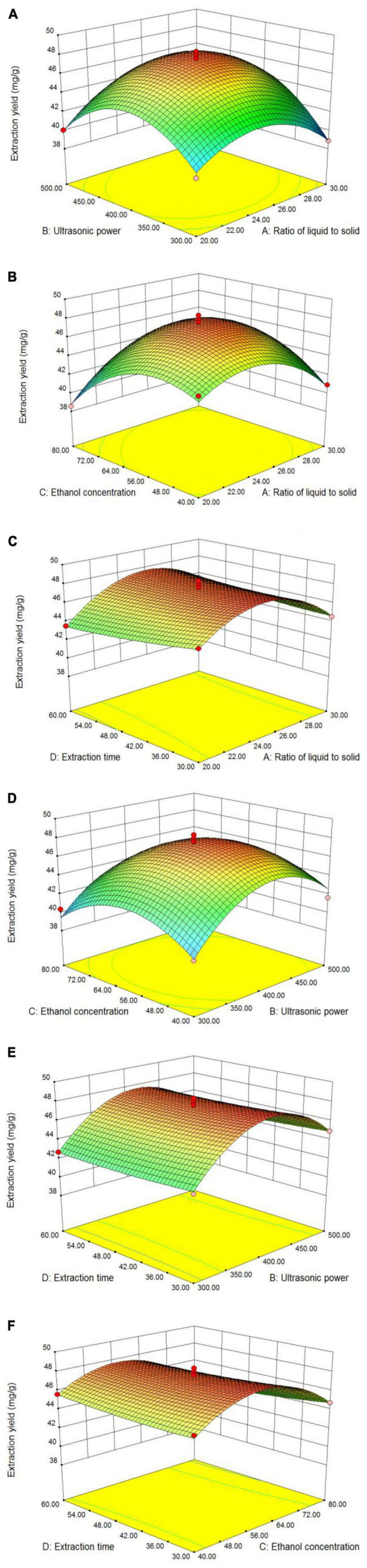
3D response surface plots showing the effect of different parameters on TFC, respectively. Ratio of liquid to solid and ultrasonic power **(A)**; ratio of liquid to solid and ethanol concentration **(B)**; ratio of liquid to solid and extraction time **(C)**; ultrasonic power and ethanol concentration **(D)**; ultrasonic power and extraction time **(E)**; and ethanol concentration and extraction time **(F)**.

The interactions of the four major parameters on the TFC are elucidated through an inspection of the three-dimensional (3D) contour plots (Figure 2), which are based on the correlation function of the independent and dependent variables (31). The maximum predicted response is located at the peak of the 3D response surface (32). In this study, the opening of the 3D response surface was downward. With an increase in the value of each of the two factors, the response value will increase. The curves in the contour map form a vertex with the increase of response surface, indicating the best TFC of LIM leaves. The steepness of the surface can reflect the influence of the investigated factors on the response value, and the greater the steepness, the greater the influence on the response value (33). The variables shown in Figures 2A, B, D had the greatest effect on flavonoids extraction, as indicated by the steep response surface curve, whereas the variables shown in Figures 2C, E, F had little effect on TFC, as indicated by the smooth response surface curve.

Thus, the influence of pairs of factors in Figures 2A, B, D on the TFC was investigated. The plots in Figure 2A illustrated the combined effects of the liquid–solid ratio and ultrasonic power on TFC. It clearly showed that when the liquid–solid ratio was increased to 25 mL/g, the TFC of the extracts increased and reached a peak value, after which a further increase led to a slow decrease in flavonoids extraction. The results showed that the TFC could be significantly improved only when the liquid–solid ratio was suitable, and that too low or too high a ratio would lead to a decrease in the extraction yield (34).

Figure 2B showed the effect of the liquid–solid ratio and ethanol concentration on TFC. With increasing liquid–solid ratio and ethanol concentration, the TFC gradually increased to its peak value, after which the TFC started to decrease with a continuous increase of those factors. The reason was that too high an ethanol concentration and too large a liquid–solid ratio leads to a change in solvent polarity and more dissolution of other impurities (22, 35), resulting in decreased flavonoids extraction.

Figure 2D showed the effect of ethanol concentration and ultrasonic power. As the ethanol concentration increased, the TFC gradually increased. However, beyond the optimun extraction time, there was no longer a significant increase in TFC. The optimisation results showed that the liquid–solid ratio of 24.76 mL/g, ultrasonic power of 411.43 W, ethanol concentration of 58.86% and extraction time of 30 min were the optimum technological parameters for flavonoids extraction. Under these optimum conditions, the theoretical TFC can reach 48.0554 mg/g. In order to ensure that the predicted value was not biased towards the actual results, the derived optimum conditions were verified by experiments. At this optimum point, the average TFC was 49.01 mg/g in three validation experiments. Thus, the actual results were close to the theoretical prediction. Therefore, the optimal extraction conditions obtained by response surface optimization were accurate and reliable, and these extraction process parameters were taken as the optimal parameter values for flavonoids extraction from LIM.

### Effect of extraction methods on the yield of flavonoids

The effect of MAE, UAE and solvent extraction on the yield of flavonoids of LIM extracts is shown in Table 4. Flavonoid yields varied depending on the extraction method. The MAE increased the flavonoid content of LIM leaves by 55% when compared to the solvent extraction, consuming the same dose (30 mg/mL) of 70% ethanol. For the UAE, less solvent was consumed and the extraction time was shorter compared to the other two methods, but the highest flavonoid content was obtained from LIM leaves. The highest extraction rate by UAE may be attributed to the strong cavitation of such forces during extraction to form bubbles, which cause damage to the cell walls of plant tissues, collapsing their surfaces and forming cracks through which bioactive compounds are extracted by the solvent (36, 37). As for the extraction of polysaccharides, Shang et al. (38) also concluded that higher polysaccharide contents were obtained with UAE and MAE compared to the solvent extraction method. In the present study, the flavonoid content obtained by UAE was significantly higher than that of solvent extraction and MAE. Therefore, it is more appropriate to use ultrasonic extraction to extract flavonoids from LIM.

**TABLE 4:** Effect of extraction methods on the yield of flavonoids.

Number	Extraction methods	Extraction time	Solvent	Solvent consumption (mL/g)	TFC (mg/g)
2	Solvent extraction	40	70% Ethanol	30	38.52 ± 1.52c
3	Microwave-assisted extraction	60	70% Ethanol	30	43.55 ± 1.89b
	Ultrasonic-assisted extraction	30	60% Ethanol	25	49.01 ± 1.64a

Different lowercase letters mean significant difference (*p* < 0.05) under the difference extraction method.

### Antioxidant activity of total flavonoids in LIM

The antioxidant properties of various plant extracts are usually attributed to their flavonoids content. The higher the flavonoids content, the greater the in vitro antioxidant capacity (39). Flavonoids can be extracted from the whole plant, as well as from the leaves, stems, roots and flowers of the plant. In our study, there were significant differences in the flavonoids obtained from different parts of LIM during different growth periods (Table 5). Overall, the total flavonoids in LIM were mainly concentrated in the flowers and leaves, with the flowers being the highest at 54.60 mg/g, which was significantly higher than the roots, stems and whole plant (*p* < 0.05). In our previous study, we showed that the main flavonoids in LIM were concentrated in the flowers and leaves and showed dynamic changes during the growth and development of the plant (3). This also indicated that the flowers and leaves are the main parts of the plant with high flavonoid content compared to the roots and stems. As for the selection of the optimal harvesting period, we found that the total flavonoids in the whole plant and leaves of LIM peaked at the flowering stage, indicating that this was the optimal harvesting period. Similar results were found in a study on the accumulation of active ingredients in *Ixeris chinensis*, where the total flavonoid content was low at the seedling stage and gradually increased as the plant grew, reaching its highest total flavonoid content at flowering (40). Photosynthesis plays a key role in plant growth and development. Plants take up carbon dioxide through photosynthesis to participate in the synthesis of their secondary metabolites (3, 41). During the process from flowering to fruiting, the temperature starts to drop, photosynthesis decreases and the total flavonoid content of *Lactuca indica* showed a decreasing trend. This result will also have an important implication for flavonoids extraction.

**TABLE 5:** TFC and antioxidant potentials in different parts of LIM.

Plant parts	Harvest time	TFC (mg/g)	DPPH (% inhibition)	ABTS (% inhibition)	OH (% inhibition)
Root	Vegetative stage	4.33 ± 0.02ij	7.25 ± 0.12hi	6.10 ± 0.09k	6.16 ± 0.18g
	Flowering stage	3.81 ± 0.01j	6.38 ± 1.73i	6.25 ± 0.48k	6.30 ± 0.21g
	Filling stage	2.87 ± 0.03k	4.81 ± 1.08j	6.71 ± 1.30k	4.75 ± 0.18g
Stem	Vegetative stage	10.33 ± 0.15e	42.64 ± 3.53d	36.10 ± 1.76f	39.31 ± 2.12e
	Flowering stage	15.91 ± 0.11g	27.30 ± 2.49f	35.95 ± 2.63f	22.08 ± 2.34f
	Filling stage	6.56 ± 0.04h	10.98 ± 1.94g	20.76 ± 1.46i	16.85 ± 1.54f
Leaf	Vegetative stage	30.74 ± 1.13c	71.46 ± 3.48c	86.43 ± 2.69c	78.84 ± 3.92b
	Flowering stage	48.51 ± 0.19b	88.22 ± 2.93b	95.48 ± 3.84a	85.23 ± 2.51ab
	Filling stage	15.98 ± 0.86e	39.76 ± 3.47d	40.21 ± 2.43e	42.38 ± 3.40de
Flower	flowering stage	54.60 ± 2.43a	91.42 ± 3.08a	92.17 ± 3.80b	88.30 ± 2.63a
Whole plant	Vegetative stage	11.68 ± 0.20f	19.56 ± 2.76e	29.16 ± 0.79h	49.32 ± 2.48d
	Flowering stage	19.37 ± 1.44d	47.43 ± 1.94d	50.78 ± 2.67d	63.03 ± 3.65c
	Filling stage	4.92 ± 0.22i	8.24 ± 1.63h	10.07 ± 1.60g	20.14 ± 1.43f

All values are expressed as mean ± standard deviation (*n* = 3). Alphabetic letters indicate the significant difference (*p* < 0.05) in a row using a one-way analysis of variance (ANOVA) and Tukey’s test. TFC, total flavonoid content; DPPH, 2,2′-diphenyl-1- picrylhydrazyl assay; ABTS, 2,2′-azino-bis-3-ethylbenzothiazoline-6-sulfonic acid assay; OH, Oxhydryl.

In general, the body’s antioxidant defence system is in balance, as is the production and scavenging of free radicals in the body. However, when too many free radicals are produced or the antioxidant defence system fails, the body’s free radical metabolism becomes unbalanced, leading to lipid peroxidation, cell damage and DNA breakage (42). It is therefore important to investigate the assessment of antioxidant function. Antioxidant assays can be classified as hydrogen atom transfer (HAT) or single electron transfer (ET) assays. In ETs-based assays, oxidants are used as indicators to monitor reactions and to measure the ability of antioxidants to reduce oxidants (43, 44). ABTS, OH and DPPH are all ET-based tests.

DPPH is a stable free radical. It is purple in organic solvents and absorbs at a wavelength of 517 nm. When antioxidants are added, some of the free radicals are removed and the absorption intensity at this wavelength is weakened, which can be used to assess the oxidation resistance of a substance (45). In this study, there were clear differences in the DPPH scavenging capacity of root, stem, leaf and flower extracts of LIM at different growth stages. Specifically, the DPPH scavenging capacity of flower and leave extracts at the flowering stage was significantly higher (*p* < 0.05) than that of the other treatments, with DPPH scavenging rates of 91.42% and 86.22%, respectively. The higher antioxidant scavenging capacity of DPPH in the flowering phase of the leaf and flower extracts of LIM may be attributed to their higher TFC.

ABTS is a chemical free radical initiator and also acts as a colour developer in the reaction. ABTS reacts with peroxidase and hydroperoxides (or reactive oxygen species) will produce a stable blue-green cation ABTS^+^. When added to a substance with antioxidant activity, it reacts with ABTS^+^ to decolourise the reaction system. The ABTS method is simple and rapid and has been widely used to determine the antioxidant activity of compounds and food samples. It is soluble in both water and organic solvents and is not affected by ionic strength, allowing the antioxidant activity of hydrophilic and lipophilic substances to be determined in a wide range of media (46). In this study, there were significant differences in the ABTS scavenging capacity of root, stem, leaf and flower extracts of LIM at different growth stages (Table 5). Specifically, the ABTS scavenging capacity of the leaf and flower extracts of LIM at the flowering stage was significantly higher than that of the other treatments (*p* < 0.05), with ABTS scavenging rates as high as 95.48 and 92.13%, respectively.

A reaction system model was established using the Fenton reaction method, and the mixture of H_2_O_2_ and Fe^2+^ was used to produce OH. Salicylic acid was then added to produce a coloured product. The product has a strong absorption at a wavelength of 510 nm. When a test substance with the function of removing OH is added to the reaction system, it will compete with salicylic acid for OH and reduce the amount of coloured products (47). In this study, the OH scavenging capacity of root, stem, leaf and flower extracts at different growth stages was significantly different (Table 5). Specifically, the OH scavenging capacity of the flower and leaf extracts was significantly higher than that of the other treatments (*p* < 0.05), with OH scavenging rates of 90.30 and 80.23%, respectively.

Antioxidant assays involve multiple reactions and mechanisms to estimate the antioxidant potential of any plant material. Unfortunately, due to the complexity of phytochemicals, there is no single method that accurately reflects the total antioxidant potential. Therefore, MS/MS characterisation is one of the key areas of phytochemical research used to calculate total phenolic compounds and their antioxidant potential.

### Composition of flavonoid compounds in LIM during different growth stages

Previously, Hao et al. (3) reported that the major flavonoids in LIM included luteolin, rutin, quercetin, luteolin-7-Q-glucoside, apigenin and kaempferol. In this study, these six flavonoids were selected for quantitative analysis. As shown in Table 6, the content of the six flavonoids varied significantly in different parts of the LIM at different growth stages. Four of the six target flavonoids in the whole plant had contents of luteolin (filling stage, 63.27 μg/mL), rutin (flowering stage, 566.45 μg/mL), luteolin-7-O-glucoside (flowering stage, 53.19 μg/mL) and apigenin (filling stage, 19.44 μg/mL). Dong (48) reported apigenin, luteolin and luteolin-7-O-glucoside in *Ixeris sonchifolia* (Bge.) Hance. In his study, luteolin-7-O-glucoside was the most abundant, whereas in our study luteolin was the most abundant. However, Huo et al. (49) found that luteolin was the more abundant flavonoid in *S. oleraceus* L., which is agreement with our results. In their study, the luteolin content ranged from 31 to 201 μg/g, with luteolin of some samples higher than our results. These variations can be explained by the variation in flavonoid class content with variety type and maturity, growing area and climatic conditions (46).

**TABLE 6:** Dynamic characteristics of six flavonoids in different parts of LIM.

Plant parts	Harvest period	Luteolin	Quercetin	Luteolin-7-O-glucoside	Apigenin	Kaempferol	Rutin
Roots	Vegetative stage	–	–	4.99 ± 0.12k	1.73 ± 0.13h	0.94 ± 0.04i	17.09 ± 4.02j
	Flowering stage	–	–	0.46 ± 0.03l	0.90 ± 0.05i	–	2.58 ± 0.22k
	Filling stage	–	–	0.88 ± 0.08l	–	–	1.74 ± 0.14k
Stems	Vegetative stage	5.15 ± 0.31i	8.45 ± 0.12g	10.46 ± 1.52i	2.45 ± 0.02g	0.55 ± 0.08j	50.16 ± 2.15i
	Flowering stage	8.72 ± 1.55f	11.32 ± 0.10d	42.23 ± 0.94g	4.53 ± 0.08f	2.48 ± 0.10f	646.86 ± 14.75b
	Filling stage	20.79 ± 2.58e	11.09 ± 0.65e	34.21 ± 2.02h	5.81 ± 0.58e	4.70 ± 0.58e	233.56 ± 8.76g
Leaves	Vegetative stage	7.34 ± 0.84g	10.32 ± 0.29f	49.73 ± 1.02e	2.40 ± 0.37g	1.82 ± 0.14g	590.87 ± 11.33c
	Flowering stage	4.49 ± 0.21j	10.29 ± 0.13f	60.41 ± 1.78c	1.64 ± 0.08h	1.32 ± 0.10h	157.02 ± 7.57h
	Filling stage	56.55 ± 0.96c	10.40 ± 0.06f	109.96 ± 9.41b	13.59 ± 0.48c	9.31 ± 0.36c	374.05 ± 5.65e
Flowers	Flowering stage	445.52 ± 9.42a	18.95 ± 0.86a	170.52 ± 9.56a	142.12 ± 4.49a	58.97 ± 0.22a	1487.90 ± 1.85a
Whole plants	Vegetative stage	6.69 ± 1.45h	7.70 ± 1.46h	29.00 ± 3.46i	2.28 ± 0.08g	1.26 ± 0.13h	344.90 ± 7.61f
	Flowering stage	29.50 ± 3.40d	11.44 ± 2.43c	53.19 ± 2.55d	10.69 ± 1.09d	5.01 ± 0.67d	566.45 ± 10.82d
	Filling stage	63.27 ± 2.61b	11.88 ± 1.80b	47.84 ± 3.69f	19.44 ± 2.48b	10.14 ± 2.48b	358.99 ± 6.37f

All values are expressed as mean ± standard deviation (*n* = 3). Alphabetic letters indicate the significant difference (*p* < 0.05) in a row using a one-way analysis of variance (ANOVA) and Tukey’s test. “–” indicates no data.

All four components were detected in different parts of LIM, except for luteolin, quercetin, apigenin and kaempferol which were not detected in the roots of LIM. We found that luteolin and luteolin-7-O-glucoside were predominantly present in the roots and stems of LIM, with the highest levels of luteolin being 172.09 μg/g and 646.86 μg/g, respectively. The highest concentrated concentration of the six flavonoids was found in the flowers of LIM, and the content of the different components was significantly different from other organs (*p* < 0.05). Rong (8) reported a higher rutin content in the leaves of LIM than in the other parts (roots, stems and whole plant), with a peak at the flowering stage, which contrasts with our results. The reason for this difference may be due to differences in growing conditions and sampling sites. Our trial sites were pre-treated with applied substrate fertiliser, whereas theirs were not. In addition, our study included the measurement of components in the flowers.

**FIGURE 3:**
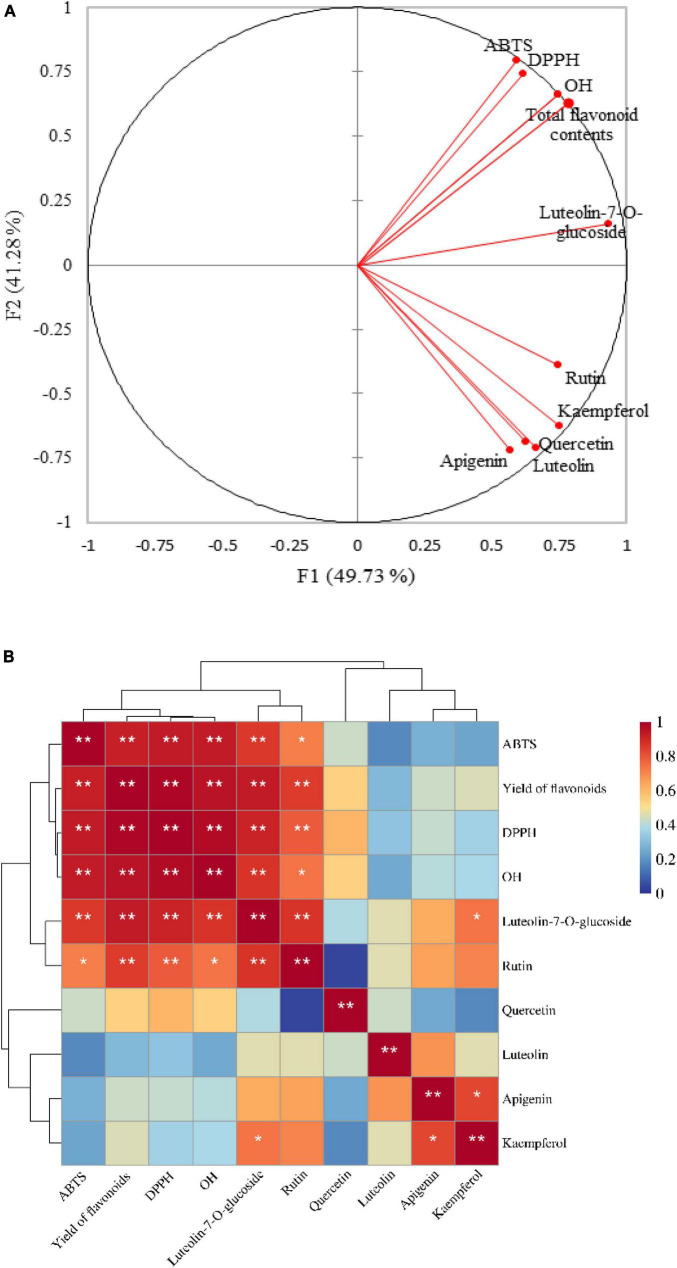
Principal component analysis (PCA) of the flavonoid content (TFC and flavonoids—quantified through UPLC-MS) and antioxidant activities (DPPH, ABTS and OH) **(A)**; correlation clustering marker heatmap between flavonid compositions and antioxidant activities **(B)**. According to the Pearson correlation coefficient, stronger correlations are shown in red and weaker in blue. *indicates significant difference at *P* < 0.05, **indicates significant difference at *P* < 0.01.

In this study, in order to explore the extent of the contribution of flavonoid components to the antioxidant properties of LIM, we performed a correlation analysis between flavonoids and antioxidant indicses (Figure 3B). In addition, principal components analysis (PCA, Figure 3A) was performed to investigate the overall similarities and differences between the flavonoid content, targeted flavonoid through UPLC/MS in different samples of LIM, and the relationship between the different methods used to evaluate the antioxidant potential.

A total of 91.01% of the variability of the original data can be explained by the first two factors (F1 and F2) in Figure 3A. For the determination of antioxidants, DPPH, ABTS and OH were strongly correlated with each other (*p* < 0.01). Floegel et al. (50) have already reported this significant positive correlation. They found that both the DPPH and ABTS assays evaluated free radical scavenging capacity. Compared to the DPPH assay, the ABTS assay can better reflect the hydrophilicity, lipophilicity. Our research also well reflects well that the flavonoids in 13 samples of LIM have strong reducing ability of DPPH and ABTS. Strong correlations (*p* < 0.01) were found between TFC and three antioxidant assays, indicating that total flavonoids in the test samples had a significant contribution to the antioxidant activities. These results are in agreement with our previously published studies on flavonoids in leaves of LIM samples and their antioxidant potential (8). Luteolin-7-O-glucoside and rutin were significantly (*p* < 0.05) or highly significantly (*p* < 0.01) correlated with all in vitro antioxidant assays, whereas kaempferol, apigenin and luteolin were not significantly (*p* > 0.05) correlated with antioxidant properties, which is inconsistent with the previously disscussed correlation between TFC value and antioxidant assays. One of the reasons may be that we only selected the six flavonoids for quantitative analysis in all LIM samples, whereas TFC assays react specifically with all types of flavonoids. Overall, the flavonoids were strongly correlated with the antioxidant assays, indicating that flavonoids have strong antioxidant activities.

## Conclusion

Our method provided complete extraction and antioxidant assay information of the flavonoids in LIM. In this study, ultrasonic-assisted ethanol aqueous solution extraction was investigated with a three-variable, three-level experiment using BBD combined with RSM to determine the highest extraction rate of flavonoid in LIM. Then, the UPLC/MS technology was used to investigate the content of the six major constituents in LIM. The data not only showed that there were differences in the active constituents of the aerial and subterranean parts of LIM, but also provided an effective reference for the dynamic selection of the best harvesting time based on the accumulation of target constituents. Flowers and leaves were the main harvested parts of LIM, and the flowering period was the most suitable harvest time. At the same time, its higher flavonoids represent a very high antioxidant potential in vitro, which has potential utility in food, feed and nutraceuticals. In the future, in vitro digestibility, bioavailability, toxicological, and animal studies will be required to develop flowers and leaves as commercial ingredients.

## Data availability statement

The original contributions presented in this study are included in the article/supplementary material, further inquiries can be directed to the corresponding author.

## Author contributions

JH contributed to methodology, visualization, validation, data curation, and wrote the original draft. ZW and YJ interpreted the data and edited the language. ZF, MZ, YL, and NY contributed to conceptualization, acquisition, reviewing, and editing. BC and LZ contributed to software. GG contributed to conceptualization and funding acquisition. All authors have read and agreed to the published version of the manuscript.
